# Nicotinic Acetylcholine Receptors Containing the α7-Like Subunit Mediate Contractions of Muscles Responsible for Space Positioning of the Snail, *Helix pomatia L.* Tentacle

**DOI:** 10.1371/journal.pone.0109538

**Published:** 2014-10-10

**Authors:** Tibor Kiss, Nóra Krajcs, Zsolt Pirger, László Hernádi

**Affiliations:** 1 Chemical Ecology and Neurobiology Group, Department of Experimental Zoology, Balaton Limnological Institute, MTA Centre for Ecological Research, Tihany, Hungary; 2 Adaptive Neuroetology MTA-CER, National Brain Project Team, Tihany, Hungary; Chiba University Center for Forensic Mental Health, Japan

## Abstract

Three recently discovered tentacle muscles are crucial to perform patterned movements of upper tentacles of the terrestrial snail, *Helix pomatia*. The muscles receive central and peripheral excitatory cholinergic innervation lacking inhibitory innervation. Here, we investigate the pharmacology of acetylcholine (ACh) responses in muscles to determine the properties of the ACh receptor (AChR), the functional availability of which was assessed using isotonic contraction measurement. Using broad spectrum of nicotinic and muscarinic ligands, we provide the evidence that contractions in the muscles are attributable to the activation of nAChRs that contain the α7-like subunit. Contractions could be evoked by nicotine, carbachol, succinylchloride, TMA, the selective α7-nAChR agonist choline chloride, 3-Bromocytisine and PNU-282987, and blocked by nAChR selective antagonists such as mytolon, hexamethonium, succinylchloride, d-tubocurarine, hemicholinium, DMDA (decamethonium), methyllycaconitine, α-Bungarotoxin (αBgTx) and α-Conotoxin IMI. The specific muscarinic agonist oxotremorine and arecoline failed to elicit contractions. Based on these pharmacological properties we conclude that the Na^+^ and Ca^2+^ permeable AChRs of the flexor muscle are nicotinic receptors that contain the α7-like subunit. Immunodetection experiments confirmed the presence of α7- or α7-like AChRs in muscle cells, and α4-AChRs in nerves innervating the muscle. These results support the conclusion that the slowly desensitizing αBgTx-sensitive responses obtained from flexor muscles are produced by activation of α7- like AChRs. This is the first demonstration of postsynaptic expression and an obligatory role for a functional α7-like nAChR in the molluscan periphery.

## Introduction

Acetylcholine (ACh), the first neurotransmitter to be discovered, is commonly distributed throughout the animal kingdom [1], [2]. Biochemical and histochemical studies in gastropod and cephalopod molluscs demonstrated the presence of ACh, the synthesizing enzyme choline acetyltransferase, and the degrading enzyme acetyl cholinesterase, in the central nervous system (CNS) [3], [4]. The effects of ACh on molluscan neurons have been shown to involve three separate receptors, one excitatory and two inhibitory, each corresponding to a specific permeability change of the surface membrane [5]–[7]. Kehoe [8] analyzed the ACh-receptor types in the pleural ganglion of *Aplysia*, concluding that the receptors mediating Cl-dependent inhibition and Na-dependent excitation do resemble nicotinic acetylcholine receptors (nAChR). Thereafter both muscarinic and nicotinic receptors have been found in the CNS of molluscs, located mainly in the neuropil, axons, and glial cells and rarely observed on the soma [9]–[11]. The AChRs cloned so far from invertebrate and vertebrate animals have revealed substantial homologies and also differences between amino acid sequences [2], [12]. At the periphery of molluscs, application of ACh has been found to produce depolarization and contraction of the anterior byssus retractor, the pharynx levator, the radula protractor, the gill, and the buccal and swim muscles [10], [13]–[21]. ACh is also the excitatory neurotransmitter at the salivary and mucus gland cells, and has either an inhibitory or a biphasic effect in the heart [15], [22]–[27]. In *Aplysia*, ACh has been shown to exert a biphasic effect on the radula closer and the parapodial muscle, acting simultaneously at hyperpolarizing and depolarizing ACh receptors [28], [29].

Taken together, the results listed above underpin the view that ACh is an important and widely distributed neurotransmitter in molluscs. However, pharmacological data from various muscles in molluscs give a confusing picture regarding the nature of the receptor. At the periphery, the ACh receptor is mostly classified as nicotinic or of mixed subtype; however the receptor classification is far from complete, because the pharmacology of the neuromuscular AChRs appears to vary considerably between different molluscan species and presumably between different muscles [19], [30], [31]. In vertebrates there are many nAChR subtypes, each consisting of a combination of 17 subunits and mediating diverse physiological functions. Vertebrate nAChRs are widely expressed in the CNS, while in the periphery they mediate transmission at the neuromuscular junction. nAChRs are also found in non-neuronal/muscle cells such as keratinocytes, epithelia, macrophages, etc. [32]. Previously, we have described that externally applied ACh at physiologically relevant concentrations is able to evoke contraction in the flexor muscles (FM) of snail tentacles. The tentacle muscles execute specific tentacle movements during olfaction thereby they are an important part of feeding, avoidance and mating behaviors in the snail [33]. Scanning of the environment by protracted tentacles, and twitching and quivering movements performed during olfactory orientation, are primarily due to the contraction of three FMs: M1, M2 and M3. The ACh effect is comparable to that elicited by electrical nerve stimulation and both responses are effectively attenuated by cholinergic antagonists [34]. It is suggested therefore that contraction evoked by the excitatory neurotransmitter ACh is due to agonist binding at specific membrane receptors, resulting in the opening of cationic (mainly Na^+^) channels, which in turn stimulates the Na-Ca exchange mechanism, thereby increasing the concentration of cytosolic Ca^2+^ entering from the extracellular space [35]. Here we examine the pharmacology of the neuromuscular contacts of FMs as a model for cholinergic transmission, since the pharmacological properties of the cholinergic response have not been investigated in detail. To address this issue, we characterized the pharmacological profile of the muscle AChR, identified the receptor subtype by Western blot and PCR, and assessed the cellular distribution by immunohistochemistry.

## Materials and Methods

### Ethics Statement

There are no ethical approvals required for the research using invertebrate animals, such as the gastropod snail *Helix* in Hungary; however every effort was made to decrease the number and the suffering of animals. Totally ∼250 snails were used. No specific permits were required for the described field collections. The animal collection site (GPS coordinates N:46^o^57,248′ and E:17^o^ 53,663′ and surrounding) is public and is not situated on private or protected land. Nevertheless the study was conducted in strict accordance with the recommendations in the guidelines for the treatment of animals of the Institutional Biosafety Regulations (VE-I-001/01890–10/2013).

### Preparation and contraction recording

Adult specimens of the pulmonate snail, *Helix pomatia*, were collected from the surrounding area. FMs of the tentacles were isolated and their isotonic contraction recorded as described previously [35]. Briefly, one end of a single muscle was fixed in a slot cut out of Plexiglas, leaving the other end free, and changes in muscle length were measured in control and test solutions using the ocular micrometer on a stereomicroscope. Agonists and antagonists were dissolved in physiological solution containing (in mM) 80 NaCl, 4 KCl, 10 CaCl_2_, 5 MgCl_2_ and 10 Tris-HCl (pH = 7.4), and were perfused onto the muscle via a rapid application device. The geometry of the perfusion chamber enabled a fast exchange of solutions (2–3 sec). Flow of solution through linearly arranged tubes was initiated by a gravity perfusion system. For the electrical stimulation of innervated muscles a pair of silver hooked electrodes was placed under the nerve. The ganglion was placed in a pit of the recording chamber and separated from the muscle by a Vaseline gap. 10 ms electrical pulses of 5–10 V were applied at 1–1.5 Hz.

### Chemicals

All chemicals used were of analytical grade. The α-bungarotoxin (αBgTx), α-conotoxin ImI (α-CTx IMI) and αA-conotoxin PIVA (αA-CTx PIVA) were purchased from Alomone Labs (Jerusalem, Israel). Acetylcholine chloride (ACh), succinylcholine chloride (Succ), nicotine hydrogen tartrate (nicotine), d-tubocurarine chloride (dTC), atropine (Atr), strychnine hydrochloride (Str), arecoline hydrobromide (Arec), tetramethylammonium chloride (TMA), PNU-282987 hydrate, benzoquinonium chloride (Myt, mytolon) and orphenadrine (Orph) were purchased from Sigma-Aldrich Co., Budapest, Hungary. The decamethylene-bis-dimethylammonium bromide (DMDA, decamethonium), hexamethylene-bis-trimethylammonium iodide (Hex, hexamethonium), carbamoylcholine chloride (carbachol) and hemicholinium-3 (Hem) are products of Fluka AG, Buchs, Switzerland. Choline chloride (ChCl) was obtained from Merck, Darmstadt, Germany. The levetimide (Lev), oxotremorine (Oxo) and scopolamine (Scop) are products of Research Biochemicals, Natick, MA, USA. RJR 2403 oxalate (RJR 2403), 3-Bromocytisine (3-BrCy), methyllycaconitine citrate (MLA) and dihydro-β-erythroidine hydrobromide (DHβE) were obtained from Tocris, Bristol, UK.

### Western blot

The CNS, FM and columellar muscle (CM) were homogenized in ice cold 50 mM Tris-HCl buffer containing 0.1% Triton X–100 and a protease inhibitor cocktail. After centrifugation at 12,000×g at 4°C for 20 min, the supernatant was collected and processed on an 8% sodium dodecyl sulfate polyacrylamide gel (SDS-PAGE). The protein concentration of samples was determined by Bradford method and the average protein content of the samples loaded on the gel was the same. After electrophoresis, proteins were blotted onto PVDF Immobilon-P membrane (Millipore). Membranes were blocked with 5% non-fat milk at room temperature and thereafter incubated overnight at 4°C with anti-nAChR α7 (ab10096,1 µg/ml, Abcam, Cambridge, UK or ANC-007, 4 µg/ml, Alomone, Jerusalem, Israel) or anti-nAChR α4 (ANC-004, 4 µg/ml, Alomone, Jerusalem, Israel) primary antibody. In preadsorption controls the proportion of antigens and their immunogens were 1∶4 (ab10096), 1∶5 (ANC-007) and 1∶2 (ANC-004). After incubation with HRP-conjugated goat anti-rabbit secondary antibody (1∶10.000, Sigma, Budapest, Hungary), the primary labeled bands were visualized with ECL substrate (WesternBright, Advansta, Menlo Park, CA, USA or Pierce ECL Western Blotting Substrate, Rockford, IL, USA).

### Immunohistochemistry

The CNS and FMs were dissected and fixed in 4% paraformaldehyde diluted in 0.1 M PB, pH 7.4) for 6 h at 4°C. Immunohistochemical procedures were carried out on 40 µm thick cryostat sections made from the cerebral ganglion (CG), as well as on whole mount preparations of the tentacle FMs. Both the sections and the whole mount preparations were incubated for 24 h at room temperature with polyclonal antibodies raised against the α4 and the α7 subunit of the nAChR (human) in rabbit. The antibodies were diluted 1∶1000 in PBS-TX containing 0.25% bovine serum albumin. After a short wash in PBS-TX the immunoreaction was visualized using the polymer HRP conjugated donkey anti-rabbit secondary antibody (One-step Polymer-HRP IHC Detection System, BioGenex, USA). Specificity of the antibodies was tested by pre-absorption with the control peptide of the antibody (Abcam). Method controls were performed by omitting the primary antibodies from the incubation solution.

### RNA Analysis

Total RNA was extracted using TRI Reagent (Sigma) from freshly homogenated CNS, CM and tentacular FM. The total RNA content was determined by calculating the ratio of the absorbance measured at 260 nm and 280 nm in spectrophotometric analysis. The RNA content of the samples were as follows: 1,2 µg/µl for CNS, 0,55 µg/µl for CM and 0,3 µg/µl for FM.

The quality and quantity of total RNA from CNS and muscles were assessed using formaldehyde denaturation gel. Reverse transcription of the isolated total RNA was performed, and the resulting cDNA was subjected to PCR, using degenerate or non-degenerate primer-pairs designed to detect nAChR subunits in Lymnaea. Primers used to generate amplicons were as follows: non-degenerate primer LnAChR A fwd: 5′-GCT AGG AAT GAC CTG GAA TGC-3′, rev: 5′-GGA ACC CAC ACC ATC TGC TTA-3′, degenerate primers LnAChR A fwd 5′-GCN MGN AAY GAY YTN GAR TGY-3′, rev: 5′-GGN ACN CAY ACN ATH TGY YTN-3′, LnAChR B fwd: 5′-WSN WSN TTY GCN ACN CAR ATG-3′, rev: 5′-GCN YTN GAY WSN ACN ATG T-3′, LnAChR E fwd 5′-MGN GGN CAR GAR CAY WSN A-3′, rev: 5′-ACN GTN TGG AAY MGN GTN TTY-3′ (all primers were purchased from Csertex, Budapest, Hungary and synthetized by Microsynth, Balgach, Switzerland). PCR reaction was performed in 41 cycles (95°C for 3 min, 95°C for 30 sec, 45°C for 1 min, 72°C for 1 min, 72°C for 10 min, store at 4°C) using a T1 thermocycler (Biometra, Goettingen, Germany). Amplified products were run on 2% agarose gel, using ethidium bromide UV detection. For DNA sizing GeneRuler 100 bp DNA Ladder Plus ready-to-use (0.1 mg/ml) was applied (Fermentas, Biocenter, Szeged, Hungary).

### Statistics

Data are presented as the mean ± S.E.M. Statistical significance was assessed in all experiments using one-sample t- test. Statistical significance was accepted when P<0.05 or P<0.01.

## Results

**Figure 1:**
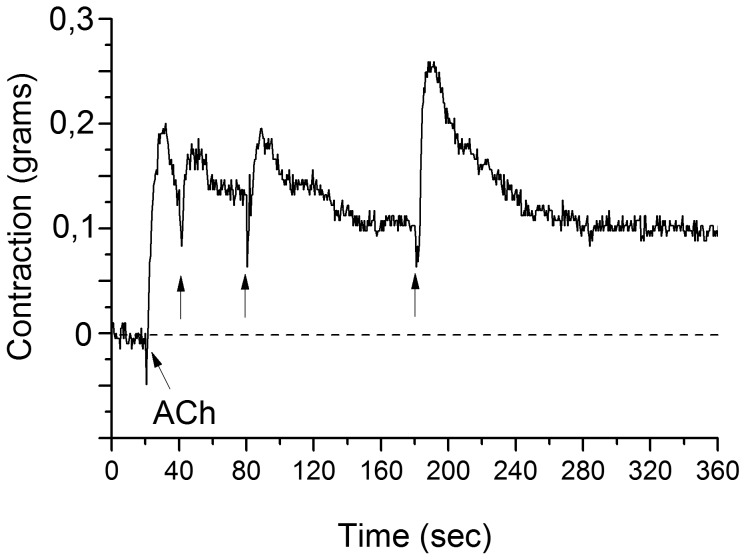
Application of 10^−4^ M ACh evoked long lasting contraction. Consecutive agonist application (arrows) did not show desensitization of the M3 muscle. The preparation was continuously perfused with physiological saline at a rate of approximately 1 ml/min. The values on the Y axis represent the force of contractions expressed in grams.

The effect of ACh on FMs of the superior tentacles was a slow and consistent tonic, concentration-dependent contracture with no phasic activity. The threshold for the depolarizing effect of ACh was between 10^−8^ and 10^−7^ M while the maximum response was observed between 10^−4^–10^−3^ M. The effective concentration of the ACh (EC_50_ = 7×10^−6^ M see [34] to induce contraction is sufficiently low to provide compelling evidence that ACh is an excitatory neurotransmitter at the neuromuscular contacts of FMs. At concentrations producing maximum contraction (10^−4^ M), repeated ACh application did not lead to a reduction in size of the response, demonstrating slow desensitization properties of the muscle AChRs (Fig. 1).

### Cholinergic agonists

**Figure 2:**
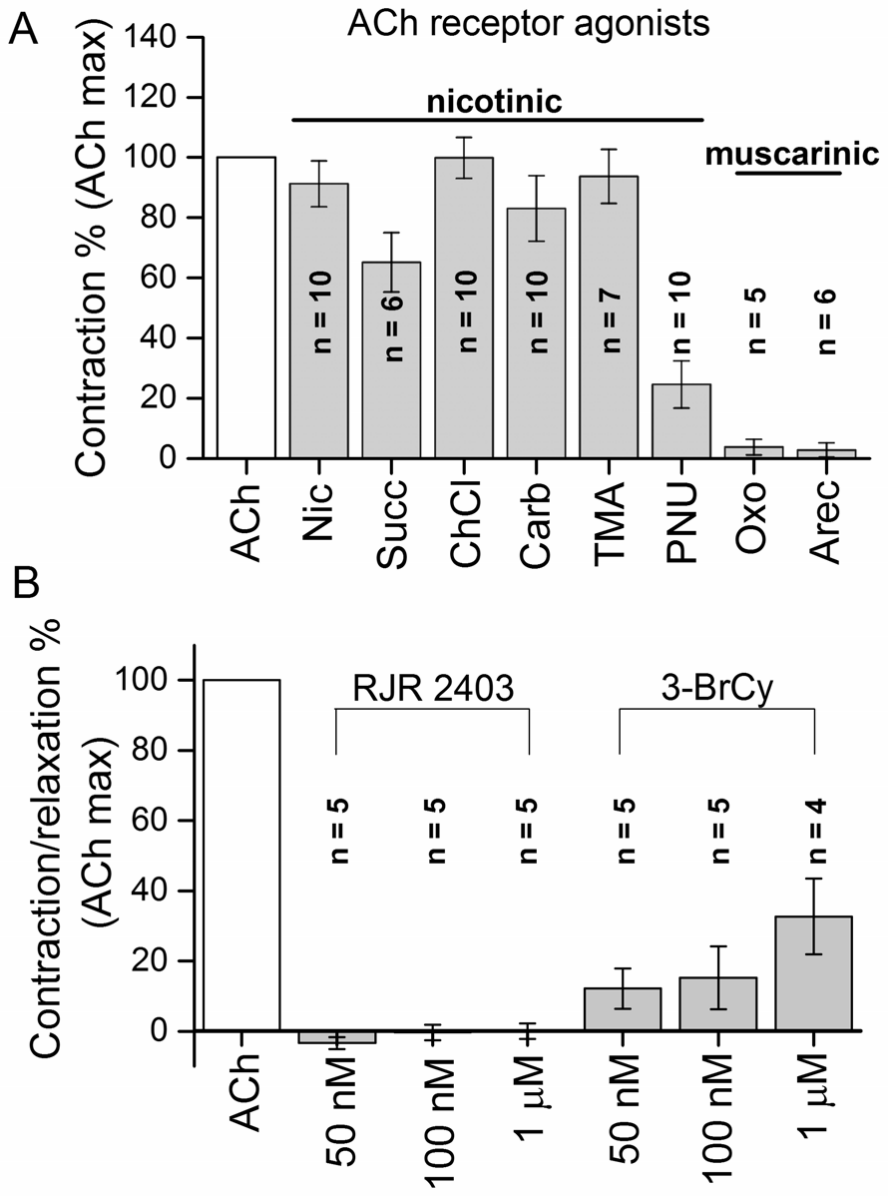
A-Effect of nicotinic and muscarinic agonists on the flexor muscle. Responses are expressed as % of the contraction elicited by 10^−4^ M ACh. Nicotinic agonist nicotine, carbachol (Carb), TMA, Succ and the α7- selective ChCl and PNU-282987 (PNU) applied at 10^−4^ M elicited muscle contraction comparable to that evoked by ACh. Muscarinic agonist Oxo and Arec applied at 10^−4^ M proved to be practically ineffective. n =  number of muscles. B-Effect of α4 and α7 subunit specific agonists on flexor muscle applied at increasing concentrations (50 nM, 100 nM and 1 µM). RJR 2403 had no effect. 3-BrCy evoked muscle contraction in a concentration dependent manner. n =  number of muscles.

A variety of cholinergic agonists were tested in an attempt to establish the pharmacological nature of the muscle AChR (Fig. 2.). The action of ACh on muscle cells was mimicked by the non-specific nAChR agonist nicotine, ChCl and TMA, and closely matched by carbachol and suxamethonium or Succ (Fig. 2A). PNU-282987, which is a drug acting as potent and selective agonist at the vertebrate homomeric α7 subtype of neural nAChR, elicited a contraction which was 25% of that elicited by ACh. 3-BrCy a potent agonist at vertebrate α7 nAChR at 31.6 nM elicited contraction which was 32% of that elicited by ACh (Fig. 2 B). The RJR 2403 had no potency to evoke contraction (Fig. 2B). The rank order of effectiveness in inducing contraction at a 10^−4^ M agonist concentration was as follows: ACh>ChCl>TMA>nicotine>carbachol>Succ>PNU-282987. The 3-BrCy at 10^−6^ M elicited contraction which was 32% of the contraction induced by external ACh. Succ is a depolarizing nAChR agonist used to induce muscle relaxation, and here elicited contraction which was 60% of that induced by ACh. Nicotine and carbachol are known to be muscle type nAChR agonists, while ChCl is a selective and full agonist at the α-BgTx sensitive α7 subunit containing nAChR. The non-selective muscarinic agonists Oxo (M2 agonist) and Arec (partial agonist on nAChR and mAChR) applied at 10^−4^ M had no potency to induce contraction (Fig. 2A). These data confirmed that ACh responses of the FM were nAChR-ionophore mediated.

### Cholinergic antagonists

**Figure 3:**
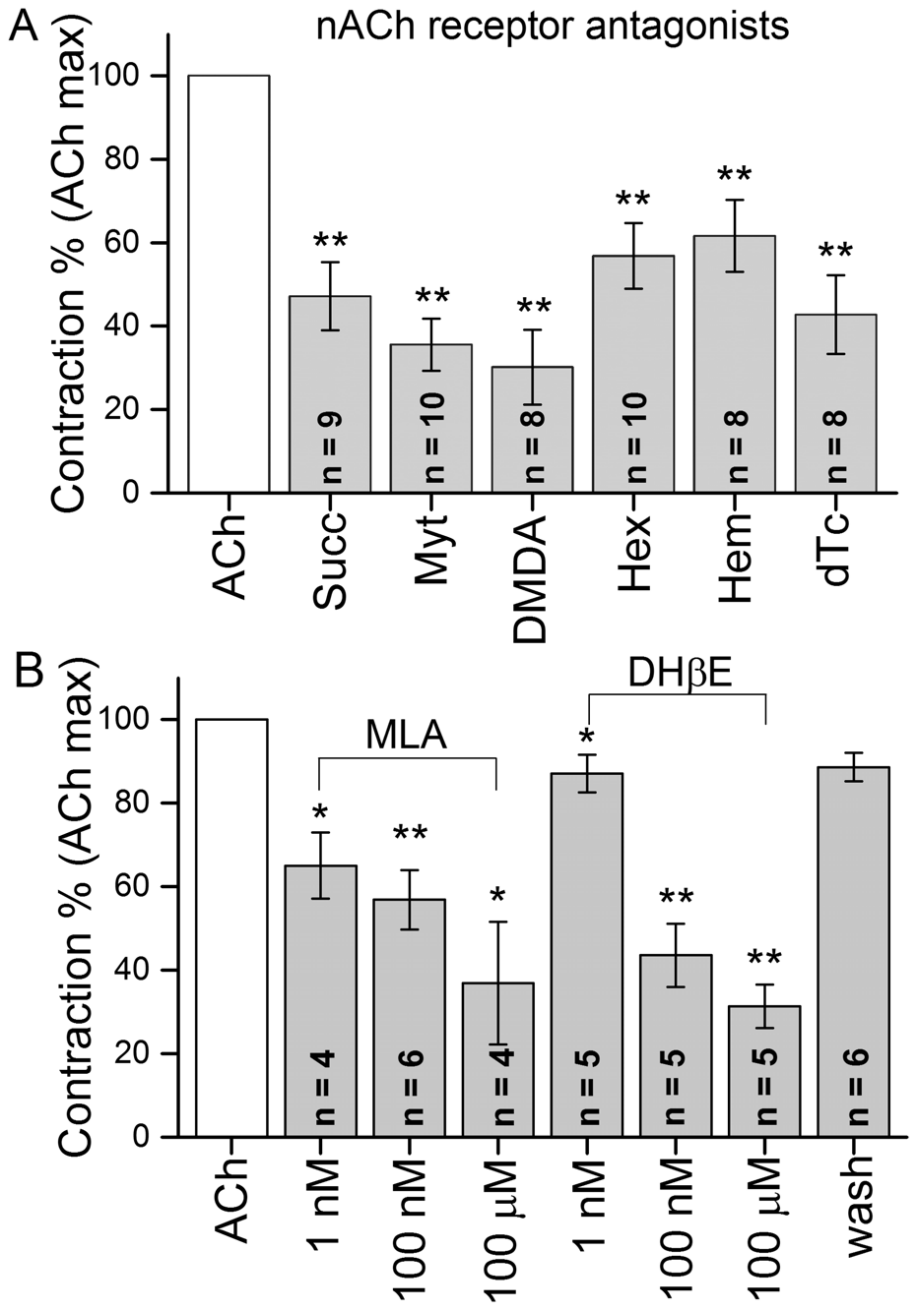
A-Relative effectiveness of nicotinic antagonists to block contractions of the flexor muscle evoked by 10^−5^ M ACh. Antagonists were applied 5 min prior to ACh administration. B – Effect of MLA and DHβE at increasing concentrations (1 nM, 100 nM and 100 µM) on 10^−6^ M ACh-evoked contractions. n =  number of muscles. Asterisks indicate a significant difference from the control value at *P<0.05, **P<0.01.

In order to characterize the receptor involved, a broad spectrum of nicotinic antagonists were tested at a 10^−4^ M concentration. The muscle cell response to 10^−5^ M ACh was rapidly (within 2 min) and reversibly reduced or blocked when the circulating bathing medium contained competitive or non-competitive antagonists such as Myt (a potent nAChR antagonist), DMDA (a peripheral ACh synthesis inhibitor that antagonizes nAChR), Succ, a depolarizing neuromuscular blocker that non-competitively binds to muscle –type nicotinic receptors, Hex, a non-depolarizing, non-specific nAChR blocker, Hem, which blocks the reuptake of choline by the high-affinity choline transporter and is classified as an indirect ACh antagonist or dTC, a competitive antagonist of several ligand gated channels (Fig. 3A). The order of effectiveness in blocking ACh elicited contraction was as follows: DMDA>Myt>dTC>Succ>Hex>Hem. The ACh-elicited contraction- blocking effects of two additional specific antagonists were tested, applied at increasing concentrations (Fig. 3B). MLA, which is a potent antagonist for α7 containing neuronal nAChRs, attenuated the contractions by 35% at 1 nM. Higher concentrations (100 nM and 100 µM) further inhibited the contractions, although at concentrations>40 nM it also interacts with α4β2 and α6β2 receptors. DHβE has moderate selectivity for the neuronal α4 receptor subunit (IC_50_ values are 0.19 and 0.37 µM for α4β4 and α4β2 receptors, respectively). At low concentration (1 nM) it attenuated the ACh-evoked contraction of FMs by 13%. At 100 nM and 100 µM it blocked ACh-elicited contractions by ∼56% and 68%.

**Figure 4:**
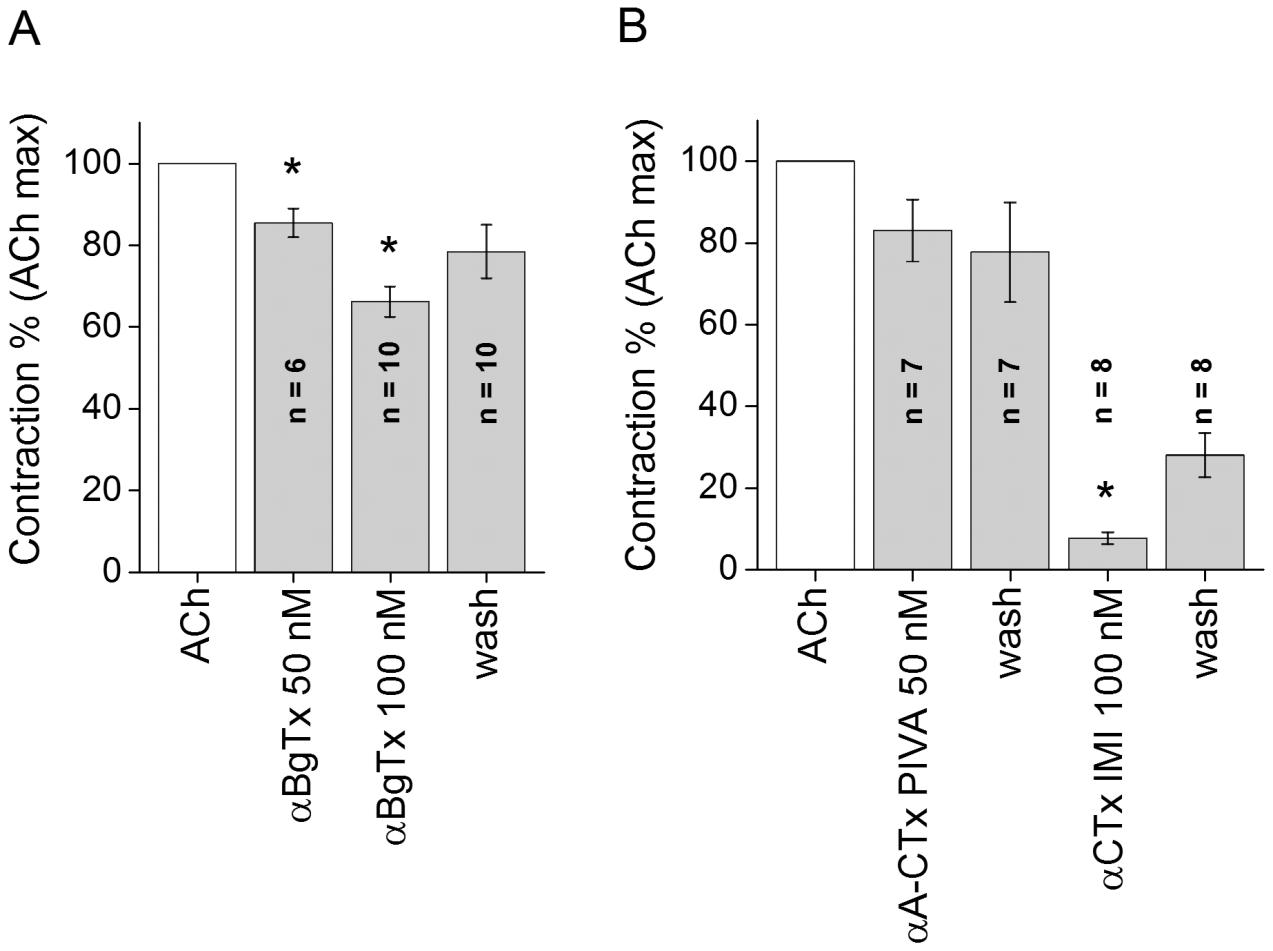
The relative effect of specific α7-nAChR antagonists to block ACh (10^−5^ M) elicited responses. A- αBgTx attenuated the ACh evoked contractions in a concentration dependent manner. The blocking effects of 50 nM and 100 nM toxin were statistically significant. The recovery is almost complete. B- The block by 50 nM αA-CTx PIVA was not significant, while the α-CTx ImI at 100 nM blocked almost completely the ACh elicited contraction. The recovery from block was partial. n =  number of muscles. Asterisks indicate a significant difference from the control value: *P<0.01.

**Figure 5:**
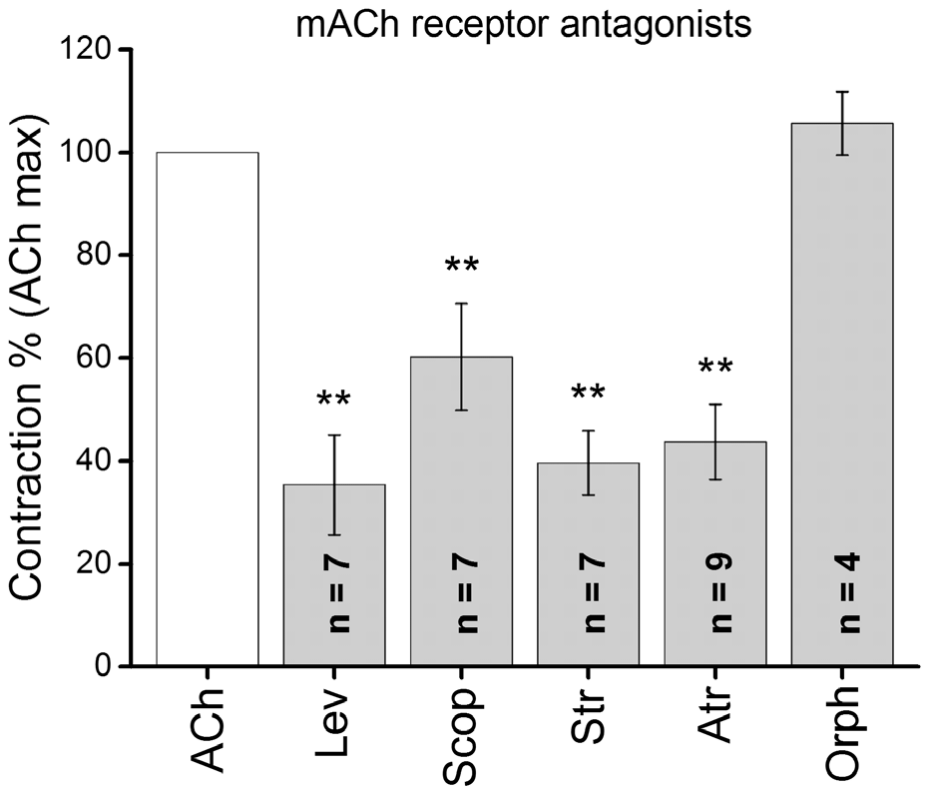
Relative effectiveness of cys-loop receptor antagonists to block ACh (10^−5^ M) elicited contractions. All antagonists were applied at 10^−4^ M 5 min prior to ACh administration. Orph, the specific mAChR antagonist proved to be ineffective. Atr is effective at all AChRs of mollusks. n =  number of experiments. Asterisks indicate a significant difference from the control value: *P<0.01.

To determine the properties of the AChR and compare with those expected for α7-nAChRs, the response of tentacle muscles was next investigated using ligands that specifically bind to the α7-subunit of the receptor. It is known that ACh, ChCl and PNU -282987 are specific activators of the α7-subunit containing nAChR. The α7-nAChR is a cation-selective, rapidly desensitizing receptor which binds α-BgTx with high affinity in mammals. The valuable property of αBgTx is that it binds to muscle nAChRs with high specificity. When αBgTx was applied at 50 and 100 nM to FMs, a concentration dependent decrease in the ACh elicited response was observed (Fig. 4A). The toxin alone showed no effect on the FMs; however, it blocked the ACh elicited muscle contraction by 34% at 100 nM. This effect was partly reversible with 80% of the ACh-elicited contraction recovering after washing the preparation for 15 min. We also examined the effect of αA-CTx PIVA and α-CTx ImI. αA-CTx PIVA is a member of nAChR-targeted *Conus* peptides. This peptide reversibly blocks postsynaptic muscle fetal α1/β1/γ/δ and muscle adult α1/β1/ε/δ nAChRs (Hopkins *et al*, 1995, Teichert *et al*, 2006); here, the αA-CTx PIVA attenuated the ACh elicited contraction by 17%. On the other hand, α-CTx ImI, a 12 amino acid peptide isolated from *Conus imperialis* venom that effectively blocks postsynaptic α7-containing nAChRs in mammal, almost completely blocked the ACh-elicited contraction (Fig. 4B). Muscarinic agonists were unable to elicit contraction of the muscle, but more surprisingly, classic muscarinic antagonists such as Atr, Lev, Scop and Str effectively antagonized contractions evoked by ACh (Fig. 5). These blocking effects were comparable to those of DMDA and Myt. However, the specific muscarinic receptor antagonist Orph proved to be inefficient in antagonizing the ACh-elicited contraction. Overall, the results of the pharmacological experiments presented here suggested that ACh-elicited contractions in the M1, M2 and M3 tentacle muscles were attributable to nAChRs containing the α7 or α7-like receptor subunit.

### The presence of α4 and α7 subunits of the nAChR in the posterior tentacles and the CG

**Figure 6:**
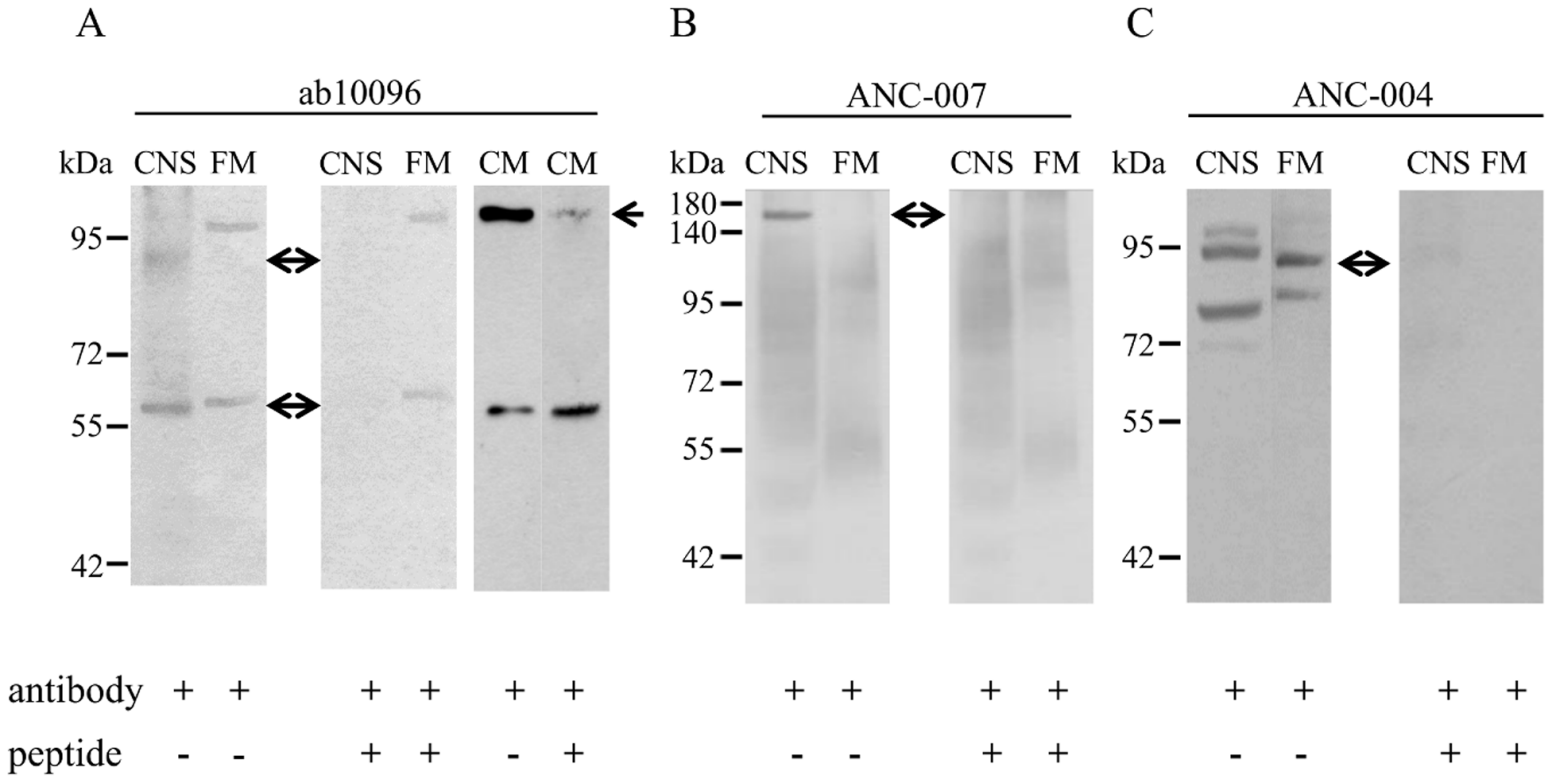
Demonstration of nAChR subtypes by western blotting using different nAChR subunit specific antibodies. A - Immunodetection of the α7 subunit in homogenates of central nervous system (CNS), flexor (FM) and columellar muscle (CM). The ab10096 recognized two bands (∼60 and 90 kDa) in the CNS which were blocked by the control peptide of the antibody (arrows). In the FM, two labeled bands (∼62 and 110 kDa) were present which could not be totally eliminated with the immunogen. In the CM, the ∼110 kDa band (but not the ∼60 kDa band) was blocked (arrow). B - Another α7 specific antibody, the ANC-007 labeled a single band in the CNS at ∼160 kDa, which was blocked with the immunogen (arrow). C – The α4 specific antibody ANC-004 labeled several bands between ∼80 and 110 kDa both in the CNS and FM, which were completely eliminated by the blocking peptide. Arrow shows the band of the size which is suggested by the producer.

The pharmacological studies described above strongly suggested that α7-containing, cation selective nAChRs participate in the regulation of the FM contraction. In order to provide additional evidence corroborating the presence and location of the α7 or α7-like subunit of the receptor, the protein extract of the FM was analyzed by probing immunoblots with several monoclonal α1-3-5-, and polyclonal α4- and α7-specific antibodies (Ab) directed against an epitope located at the extracellular N-terminal domain of human nAChR. No positive reaction was obtained with the monoclonal Ab. When using the α7 Ab (ab10096), ∼60 and 90 kDa bands were observed in the CNS homogenates which were completely eliminated by preincubating the Ab with the appropriate blocking peptide. Using the same Ab, ∼62 and 110 kDa bands were labeled in FM homogenate and they were reduced to a faint appearance with the immunogen but not blocked completely. In the CM sample similarly to FM, ab10096 labeled two bands (∼60 and 110 kDa) and the 110 kDa band could be almost fully eliminated with the immunogen (Fig. 6A). We tested another anti α7 Ab (ANC-007) purchased from a different producer, to see if there is difference in their specificity. ANC-007 labeled one single band (∼160 kDa) only in the CNS homogenate, which could however be completely eliminated with the blocking peptide. In FM homogenate no labeled band(s) was observed (Fig. 6B). Using the α4 Ab (ANC-004), positive reactions were observed as multiple bands in the CNS (∼72, 80, 93 and 97 kDa) and FM (∼85 and 90 kDa) samples, and all bands were blocked with the immunogen. The ∼90 kDa band observed in the FM is the mass, which is also suggested by the Ab (Alomone) producer for α4 subunit (Fig. 6C).

**Figure 7:**
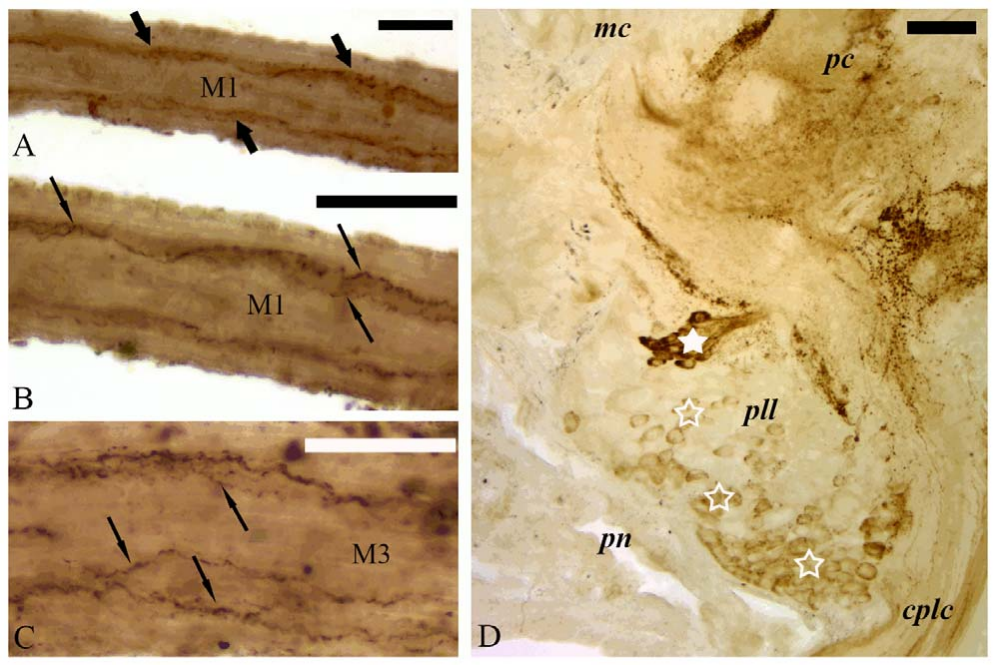
Distribution of ACh receptors containing the α4 subunit of nicotinic ACH receptor in the flexor muscles (A–C) and the cerebral ganglion (D). The α4 immunoreactivity is confined to neuronal elements in both the flexor muscles and the ganglion. In the flexor muscle M1 (A) trunks of axons (thick arrows) run along the longitudinal axes of the muscles. In the nerve trunks (B–C) fine immunolabeled axons could be seen (thin arrows). In the cerebral ganglion (D) the majority of labeled cell bodies is located in the pleural lobe (pll) and is gathered in loose groups (empty asterisks). Compact groups of small neurons can also be observed (asterisk). mc- mesocerebrum, pc- procerebrum, cplc- cerebro-pleural connective, pn- perineurium. bar: 50 µm.

**Figure 8:**
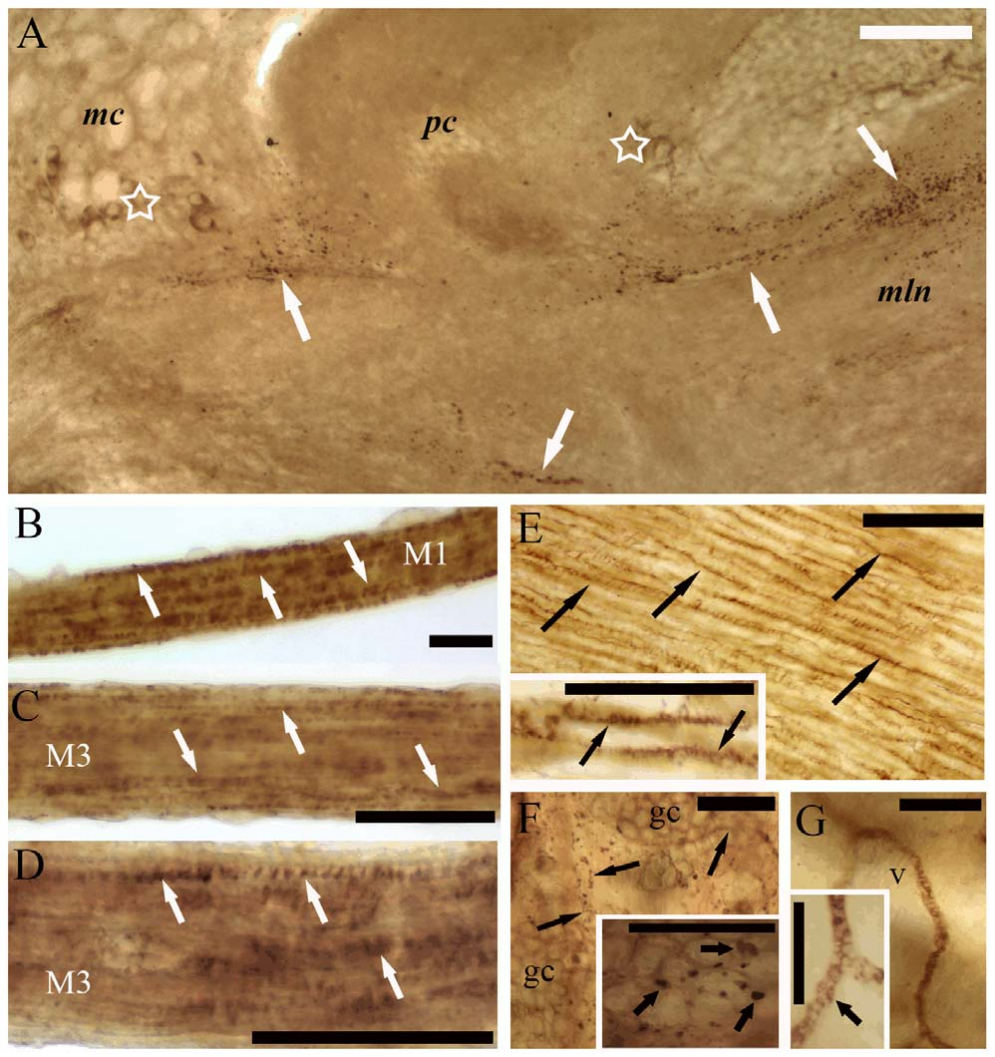
Immunoreactivity revealed by α7 subunit Ab in the cerebral ganglion (A), M1, M3 flexor muscles (B–D), and the columellar muscle (E) as well as in the stem of tentacle (F–G). In the cerebral ganglion (A) immunoreactivity can be seen in groups of small neurons (empty asterisks) in the mesocerebrum (mc) and procerebrum (pc). Additionally a few of immunolabeled varicose fibers (arrows) can also be observed. In the flexor muscles (B) immunoreactivity is distributed on thin muscle fiber trunks (arrows) and displayed dotted appearance (arrow heads) (B–C) which is a sign of the postsynaptic localization. In the columellar muscle (E) immunoreactivity is distributed on the surface of muscle fibers (black arrows). The immunoreactivity on the sarcolemma displays dotted appearance (insert, arrow heads). In the stem immunoreactivity (arrows) is located on the gland cells (gc) (F) and the wall of haemolymph vessels (v) (G). In both cases the immunoreactivity (arrow heads) shows dotted appearance (see inserts F, G). bar: 50 µm.

**Figure 9:**
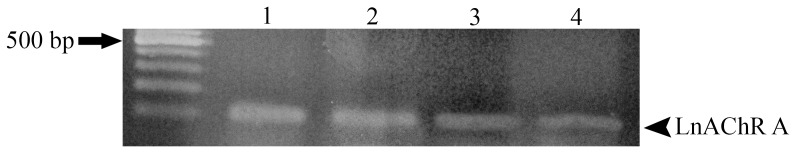
PCR products of LnAChR A subunit expressed in different tissues of snail. Left - DNA ladder, lane 1 - Lymnaea CNS, lane 2 - Helix CNS, lane 3 - Helix columellar muscle, lane 4 - Helix flexor muscle, loaded on ethidium bromide stained gel. In all lanes the calculated weight of the amplified product was ∼120 bp. DNA ladder lane shows bands at 100 bp increments. The template cDNS in the following amounts were added: 1 µl CNS cDNS, 2 µl CM cDNS and 4 µl FM cDNS in order to maintain the same volume for the PCR reaction. Accordingly, the amount of Master Mix was decreased proportionately.

Immunohistochemical experiments further demonstrated that α4 Ab immunoreactivity was mainly confined to neuronal elements in both the tentacle muscles and the CG. In the tentacles, a positive immune response was found in axonal fibers innervating the FMs (Fig. 7A, B, and C), and in the CG (Fig. 7D). In the muscles, immunolabeled axons could be seen running along the muscle fibers, frequently displaying a varicose appearance (Fig. 7A–C). In the CG, medium diameter immunolabeled cell bodies were observed on both the ventral and dorsal surfaces. The majority of the medium diameter neurons were located to the procerebrum and to the pleural lobe of the ganglion. Groups of small diameter neurons were also identified in the pleural lobe. In addition, separated labeled nerve trunks were seen in the neuropil area of the ganglion (Fig. 7D). The α7 Ab revealed positive immunoreactivity in non-neuronal elements in the tentacles, but also in a few neuronal elements of the CG (Fig. 8). In whole-mount preparations made from FMs the immunoreaction was located to the muscle fibers, displaying a dotted appearance along the FM and CM at higher magnification (Fig. 8A–D). In the tentacle stem, immunoreaction could be identified in the CM (Fig. 8 E), at the base of gland cells (Fig. 8 F) and in haemolymph vessel walls (Fig. 8 G) suggesting a wide distribution of the α7-nAChR subtype at the periphery. To further demonstrate the existence of α7 or α7-like nAChR in FM and verify the specificity of the Abs, experiments were conducted to test for the presence of α7-subunit in the CNS and in the FMs. Fig 9. shows the results of a representative PCR-experiment using non-degenerating primer pair for the amplification of LnAChRA subunit. When degenerating primers for the amplification of the LnAChR B and E (which displays homology to the vertebrate α4 subunit) subunits were used cDNA products could not be visualized. Thus PCR results confirmed the existence of LnAChR A in FM, CM and brain tissues of the snail underpinning evidences obtained by pharmacological and molecular data.

## Discussion

### The nature of the AChR is nicotinic

Previously we have provided convincing evidence that ACh is an excitatory transmitter mediating FM contractions in superior tentacles [34]. ACh released from axons of cholinergic neurons activated receptors which proved to be mainly nicotinic. The FM receptors of the snail tentacle were accessible and sensitive to externally applied ACh and nicotinic receptor agonists. Nicotine, TMA, 3-BrCy and ChCl were as potent as ACh. PNU-282987 (at 10^−4^ M) the specific α7-subunit containing AChR agonists, elicited contraction which was 25% of that of elicited by ACh. The muscarinic agonist Oxo and Arec were unable to elicit contraction confirming that the AChR is nicotinic. The ACh elicited contraction was antagonized by anti-nicotinic cholinergic drugs such as Myt, Hex, DMDA, Succ, Hem and dTC. Further evidence for a nicotinic type AChR was obtained by using specific anti-nicotinic toxins; from the nicotinic antagonists tried, αA-CTx PIVA attenuated the ACh responses, while αBgTx and α-CTx ImI potently blocked the ACh-evoked contraction of FMs. The receptor characterization was confused, however, by the ability of anti-muscarinic antagonists such as Atr, Scop, Str and Lev to block the contraction evoked by ACh. The non-specific muscarinic receptor antagonists Lev and Str, and also dTC, do block other cys-loop ligand gated channels in molluscs and probably act at the level of an ionophore common to a number of receptors, questioning their usefulness in characterizing receptor type [36]. At the same time, the specific muscarinic receptor antagonist Orph did not antagonize the ACh-elicited contraction. Previously it was described that neurons of the snail brain have nicotinic or muscarinic receptor properties in response to various cholinergic agonists. It was concluded that it is not possible to classify cholinergic receptors on the basis of antagonist action alone, at least in molluscs. Based on the almost equal sensitivity to both nicotinic and muscarinic antagonists, it appears that the muscle excitation is mediated by AChRs with mixed pharmacology [31], [37]–[39]. Such a conclusion is seemingly restricted only to lower molluscs, because binding experiments made recently on the cuttlefish nervous system questioned the existence of muscarinic-like receptors [9].Only a few studies have examined the detailed pharmacology of ACh-induced responses and receptor subtypes in various molluscan muscles. For example, the branchial heart of the cephalopod *Sepia officinalis* appears to be controlled by the αBgTx sensitive nAChR. Oxo and other muscarinic agonists do not cause diastolic arrest of the heart [40]. The inhibitory effects of Hex on the cholinergic responses of buccal muscles indicate that the receptor is related to nicotinic receptors [14], [41], [42]. Hex and dTC are inhibitors of vertebrate nicotinic receptors; however, they are more effective at inhibiting ganglionic than neuromuscular nicotinic receptors. The cholinergic response of *Aplysia* buccal muscles is also inhibited by muscarinic receptor antagonists, indicating that the cholinergic receptor in buccal muscles has a remarkably different receptor site from vertebrate cholinergic receptors, or that the muscles contain both nicotinic-like and muscarinic-like receptors. For example Ajimal and Ram [43] found that cholinergic contractions of *Aplysia* rectum could be blocked by both Atr and Hex and also that both agents cause contraction of the esophagus. However, when Hex and Atr were applied together the ACh response was not completely blocked, and the nicotinic antagonist mecamylamine did not leave any unblocked response, as might have been expected if a second type of receptor were mediating a portion of the response. In the current study, the receptors examined were activated exclusively by nAChR agonists and are thus different from those observed previously in the snail brain and muscles. Our results indicate the expression of only one nAChRs subtype in snail FM that is αBgTx sensitive, slowly desensitizing receptor. However muscarinic as well as non-cholinergic antagonists also inhibit the receptor function, distinguishing the tentacle muscle receptor from those of vertebrates. The rank order of agonist potency obtained in this study differs from that found on α7-nAChRs of vertebrates and α7- like rapidly desensitizing receptor of *Aplysia* [44], [45]. Kehoe and McIntosh [45] described three distinct nAChRs mediating two Cl-dependent and one cation-dependent cholinergic response in *Aplysia* neurons. One Cl-dependent ACh-response is rapidly desensitizing and is blocked by α-CTx ImI. The other Cl-dependent ACh-response, which is slowly desensitizing, is unaffected by the toxin and is antagonized by Hex. With the exception of α-CTx ImI, the receptors revealed similar pharmacology because they were blocked by αBgTx, dTC, DhβE, Str, MLA and activated by cytosine, nicotine and suberyldicholine. It appears that AChRs in *Lymnaea* neurons have common features with cation-selective vertebrate α7-nAChRs and one type of *Aplysia* Cl-conducting receptor [46]. The FM nAChR studied here revealed also similarities to α7-nAChRs of rat intracardiac and superior cervical ganglion neurons, which also desensitize slowly and are reversibly blocked by αBgTx. The AChR of the snail tentacle muscle shares strong pharmacological properties with the αBgTx- and α-CTx ImI-sensitive, α7 containing cation permeable receptor that mediates a rapidly desensitizing response in vertebrate neurons. The only exception was that the FMs of the snail tentacle did not desensitize, or did it very slowly.

### Nicotinic AChRs desensitize slowly and are blocked by αBgTx

The results presented here provide the first evidence that ACh-evoked contractions in the FMs of the snail are attributable to nAChRs containing a α7- or α7-like subunit. The α7-nAChR is the only subunit to be activated by the endogenous ligands ACh, 3-BrCy, PNU-282987 and ChCl. The latter was as potent as ACh, TMA and nicotine in eliciting contraction in the FMs. ChCl has received attention because it is a selective and full agonist at αBgTx–sensitive, α7 subunit containing nAChRs in vertebrates [47]. αBgTx is a potent and selective antagonist that binds with high affinity to vertebrate muscle and neuronal α7-, α8- and α9-containing nAChR [48]. Two groups of nAChRs exist, those that bind αBgTx with high affinity and for those that bind with low affinity. The latter group of receptors is found in the CNS of vertebrates and is referred to as neuronal nAChR. Neuronal nAChRs possess a higher permeability to Ca^2+^ than muscle type nicotinic receptors and are more sensitive to ganglionic blockers [49]. Invertebrate nAChR genes isolated to date all show a greater homology to the vertebrate neuronal type nAChR than the muscle nAChRs [2]. Pharmacological studies of cholinergic neuromuscular contacts in molluscs have indicated a similarity with neuronal-neuronal synapses [29].

By using degenerate PCR cloning several nAChR subunits were identified from the CNS of *Lymnaea stagnalis*, which formed functional receptors responding to ACh when expressed in oocytes. These subunits were divided into two groups according to their ability to conduct cations or anions: the LnAChR A and LnAChR B subunits. The cation conducting LnAChR A is a functional homopentameric receptor when expressed in oocytes, which can be activated by nicotine and choline and is sensitive to αCTx ImI and αBgTx [50]. The pharmacological properties of the FM nAChR described here display similarities to the *Lymnaea* LnAChR A and to vertebrate α7 subunit-containing receptors.

The presence of the α7- or α7-like AChR subunit in the snail tentacle muscles is not surprising because it is thought that this subunit gene is closest to the ancestor genes that existed millions of years ago before separation of vertebrate and invertebrate lineages [40], [51], [52]. For example the simple nematode, *Caenorhabditis elegans*, possesses the most extensive known gene family of nAChR-like subunits and shows the greatest similarity with nAChR subunits of both invertebrates and vertebrates [53]. In addition the functional and pharmacological properties of the gene product are also conserved, so as to form homopentamers with high Ca-permeability and αBgTx sensitivity. However the nAChR in the snail FMs desensitizes slowly and also conducts Na^+^ along with Ca^2+^, suggesting that the receptor is not a homopentamer [35]. Unfortunately, the Western blot experiments gave dubious results revealing several peptide bands in the range of 40–140 kDa. It is described that certain commercially available α7- antibodies lack specificity because they give the same immune response in wild-type and knock-out mice although RNA analyses confirmed the disruption of the α7 allele and lack of α7 message in the knockouts [54], [55].

Obscured data were compensated by our PCR experiments where the presence and expression of the *Lymnaea*-type (LnAChR A) nAChR subunit was assessed by PCR. PCR profiling detected expression of the LnAChR A subunit in the FM and CM and also in CNS of the snail in support of our pharmacological and immunological data. Interestingly, in the snail the α7 subunit containing nAChRs were observed mainly on muscle cells. The α4 subunit containing AChRs were detected in axons innervating the FMs, suggesting their involvement in possible feedback control of the ACh release from nerve terminals.

## Conclusions

In conclusion, our work for the first time shows the existence of α7-like nAChR in FM of the snail tentacle, leading to the idea that postsynaptically α7-like nicotinic receptors may physiologically contribute to cholinergic transmission at the neuromuscular junction in molluscs. Based on pharmacological properties we conclude that the nACh receptors of the FM are cation permeable, slowly desensitizing nicotinic receptors that are sensitive to αCTx ImI and less sensitive to αBgTx and can be activated by nicotine, 3-BrCy, TMA and ChCl. Immunodetection experiments confirmed the presence of α7-like AChRs in muscle cells, and α4-AChRs in nerves innervating the muscle. Pharmacological and immune data were further supported by the presence of transcript in the FM coding for the LnAChR A subunit as assessed using PCR assays. We conclude that the nAChRs in FM of the snail tentacle more closely resemble a LnAChR A and vertebrate neuronal α7-AChRs receptor than a muscle receptor. This is the first demonstration of postsynaptic expression and an obligatory role for a functional α7-like nAChR in the molluscan periphery.
